# Homologous recombination pathway alterations in basal-like breast cancer

**DOI:** 10.3389/fonc.2026.1772663

**Published:** 2026-06-18

**Authors:** Simon Aho, Boris Guyot, Véronique Maguer-Satta

**Affiliations:** 1 Department of medical oncology, Centre Léon Bérard, Lyon, France; 2 Cancer Research Center of Lyon (CRCL), Inserm U1052-Centre National de la Recherche Scientifique (CNRS) UMR5286, Centre Léon Bérard, Lyon, France

**Keywords:** basal like breast cancer, biomarker, homologous recombination (HR), RAD51 foci, triple negative breast cancer

## Abstract

Breast cancer is a heterogeneous disease comprising multiple molecular subtypes with distinct biological and clinical features. Among them, the basal-like subtype accounts for approximately 15–20% of cases and is associated with poor prognosis. Basal-like breast cancers (BLBC) share several histo-molecular characteristics with BRCA1-deficient tumors, including genomic instability and reduced BRCA1 expression beyond germline-mutated cases, suggesting potential alterations in homologous recombination (HR) DNA repair. These observations have led to the hypothesis that homologous recombination deficiency (HRD) may be enriched in a subset of BLBC. However, the extent, causes, and clinical implications of HRD in this subtype remain incompletely defined, as much of the available evidence is derived from triple-negative breast cancer (TNBC) cohorts, which only partially overlap with BLBC. In this review, we first summarize the transcriptomic definition of BLBC and its relationship to TNBC. We then provide a concise overview of current HRD detection methods, followed by a critical analysis of their application specifically to basal-like tumors. We next examine the molecular mechanisms that may contribute to HRD in BLBC, distinguishing well-supported alterations from emerging mechanisms. Finally, we discuss the therapeutic implications of HRD in BLBC, highlighting current limitations of clinical evidence and the challenges of translating a biologically defined subtype into a clinically actionable framework. Overall, while a BLBC-centered approach may offer a more biologically homogeneous context to study HRD, its clinical relevance remains to be established, and future studies specifically designed in molecularly defined cohorts will be required to clarify its diagnostic and therapeutic value.

## Introduction

Breast cancer is a heterogeneous disease encompassing multiple biological subtypes with distinct clinical behaviors and therapeutic responses. Among these, basal-like breast cancers (BLBC) were initially defined in the early 2000s through gene expression profiling as part of the intrinsic molecular classification. These tumors are characterized by the expression of basal cytokeratins, high proliferative activity, marked genomic instability, an overall aggressive clinical course, and a frequent triple-negative phenotype defined by the absence of estrogen receptor (ER), progesterone receptor (PR), and HER2 expression (1–3). Nevertheless, while BLBC largely overlap with triple-negative breast cancers (TNBC), the two entities are not synonymous. Approximately 70–80% of TNBC are basal-like (4, 5), whereas a subset of BLBC may not fulfill the triple-negative definition (2, 6, 7). More broadly, TNBC represents a biologically heterogeneous group of tumors (8–10), which limits its use as a surrogate for basal-like disease. Homologous recombination deficiency (HRD) refers to the inability of tumor cells to accurately repair DNA double-strand breaks through the homologous recombination repair pathway. Homologous recombination is a high-fidelity DNA repair mechanism that plays a central role in maintaining genomic integrity, notably through the coordinated activity of proteins such as BRCA1 and BRCA2. In particular, BRCA1 is involved in the detection of DNA damage, end resection, and recruitment of homologous recombination repair complexes. Defects affecting this pathway lead to genomic instability, accumulation of chromosomal aberrations, and reliance on alternative, error-prone DNA repair mechanisms (11). HRD has attracted major interest in oncology because it may confer increased sensitivity to DNA-damaging agents, particularly platinum salts and poly(ADP-ribose) polymerase inhibitors (PARPi) (12, 13). The hypothesis that HRD may play a central role in BLBC emerged from the striking similarities between BRCA1-mutated breast cancers and sporadic basal-like tumors at the clinical, histopathological, and molecular levels (14). These similarities include high-grade histology, frequent TP53 mutations, characteristic patterns of genomic instability, and shared gene expression profiles. Together, these observations led to the concept of “BRCAness,” referring to tumors that share molecular and functional characteristics of BRCA1/2 deficiency in the absence of identifiable germline mutations (15–17). Subsequent studies further supported the idea that HRD-related alterations are enriched in basal-like tumors through a variety of mechanisms extending beyond BRCA1/2 mutations alone, including epigenetic, transcriptional, and microenvironmental processes (18–25). Over the past decade, numerous studies have investigated HRD in breast cancer using a wide range of approaches, including chromosomal aberration–based scores, mutational signatures, transcriptomic profiling, and functional assays such as RAD51 nuclear foci formation (26). These studies generally suggest enrichment of HRD-related features in basal-like tumors and raised the possibility that HRD assessment could help identify tumors more likely to benefit from platinum-based chemotherapy or PARP inhibitors (20, 22, 27–33). However, despite this strong biological rationale, the clinical implications of HRD in BLBC remain incompletely defined. Unlike in ovarian cancer, where HRD assessment has become an important component of therapeutic decision-making (34–36), the predictive value of HRD in breast cancer remains more controversial. While several studies have reported improved responses to platinum salts or PARP inhibitors in HRD positive TNBC and basal-like–enriched populations (27, 37–44), the magnitude and consistency of benefit appear more heterogeneous, particularly in the metastatic setting (45–50). In addition, functional studies suggest that only a subset of basal-like tumors exhibit true homologous recombination deficiency despite frequent genomic HRD-associated alterations (24, 29). This discrepancy likely reflects the biological complexity and temporal dynamics of HRD, as genomic scar–based approaches capture historical DNA repair defects whereas functional assays evaluate current homologous recombination activity. Another important limitation is that relatively few studies have specifically focused on BLBC defined by intrinsic subtyping. Instead, most available data derive from TNBC cohorts, from which basal-like–specific conclusions are often inferred. Given the biological heterogeneity within TNBC, this may obscure subtype-specific patterns of HRD prevalence, mechanisms, and therapeutic relevance. In this review, we examine basal-like breast cancers through the specific lens of homologous recombination deficiency. We discuss the biological rationale supporting HRD enrichment in BLBC, review the main methods currently used to assess HRD in this context and their limitations, and explore the diverse mechanisms potentially responsible for HRD in basal-like tumors. Finally, we discuss the therapeutic implications of these findings and address whether intrinsic basal-like classification may provide added value over TNBC as a framework for study ing HRD biology and refining future clinical strategies.

## Basal-like breast cancers: what are we talking about?

### Transcriptomic definition and surrogate markers in immunohistochemistry

In the 2000s, a succession of transcriptomic analysis identified 5 distinct breast cancer subtypes called intrinsic subtypes: luminal A, luminal B, normal-like, HER2 enriched and basal-like (1–3), the latter being associated with the worst prognosis in terms of age at diagnosis, overall survival and disease-free survival (6, 51–53). From a transcriptional point of view, BLBCs are so named because they robustly express a cluster of genes also expressed by cells located in the outer (basal) layer of the mammary gland: myoepithelial cells as well as stem cells and basal progenitors (1–3, 54). Some of these genes products, in particular cytokeratin 5 (CK5) and cytokeratin 17 (CK17), but also laminin, integrin-β4 or even BPAG1 are normally involved in the anchoring of basal cells to the basal membrane (55). Nevertheless, beyond this structural role, some are also involved in various tumorigenic signaling pathways, in particular ERK, PI3K-AKT or NFκB (56). Other basal-like gene products are involved in epithelial-mesenchymal transition (EMT) (TGFβ2, MMP14, TM4SF1), proliferation (cyclin E1, BUB1, EZH2) or even stemness (cKIT, TGFβ2) (57). In contrast, among the least expressed genes are genes coding for Erα, PR and HER2, BLBC being predominantly triple-negative. As transcriptomic analysis are not easily feasible in current practice, several teams sought to develop panels of immuno-histochemical markers to reliably identify BLBC. Historically, the triple-negative phenotype was used for this purpose. However, not all TNBC are basal-like and a minority of BLBC are not triple-negative either (2, 6, 7). Thus, additional markers, comprising in particular basal cytokeratins (CK5/6, CK14, CK17) and EGFR, were used for better discrimination of cases. For example, Nielsen et al. established that a panel of 4 markers (negativity of Erα and HER2 associated with positivity of CK5/6 and/or EGFR) makes it possible to discriminate BLBC (defined according to the intrinsic classification) with a 76% sensitivity and a 100% specificity (4). This classification is the most commonly used in translational research. Additionally, Livasy et al. studied the IHC profile of previously selected BLBC based on their gene expression profile (58). The most frequently encountered immunophenotype was Erα and PR negativity associated with CK5/6, CK8/18, EGFR and vimentin positivity. This positivity of the luminal markers CK8/18, associated with the frequent negativity of the myoepithelial markers SMA, p63 and CD10 is interesting. It makes the hypothesis of a differentiated myoepithelial origin for BLBC unlikely, but rather suggests the involvement of a bipotent progenitor, as mentioned by several other studies (8, 59–61). Other groups have integrated additional markers into their diagnostic panel, thus increasing their specificity at the cost of sensitivity. They are comprehensively described in the review by Choo and Nielsen (62) and Botti et al. (63).

### Additional classifications

**Figure 1:**
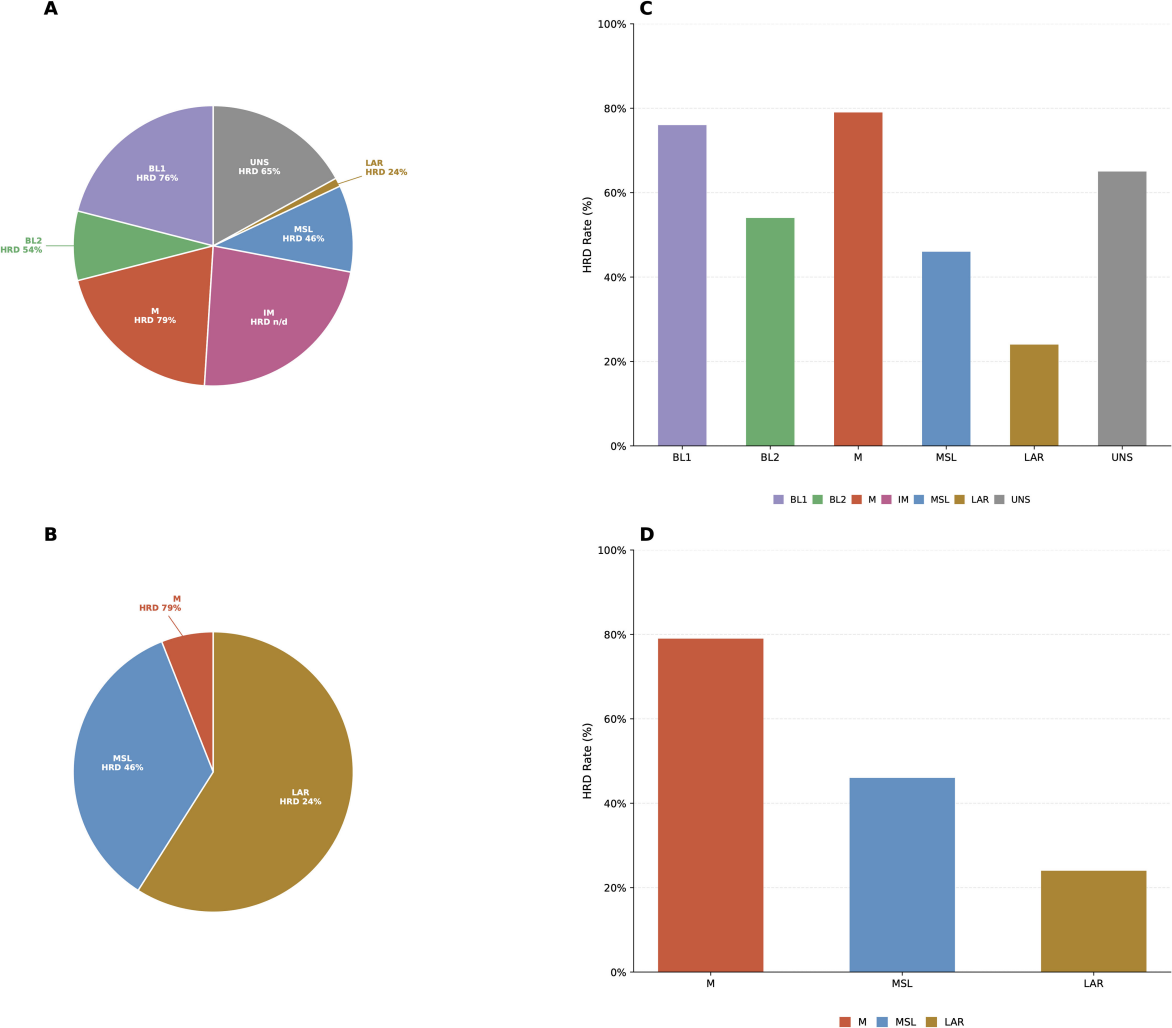
(From Masuda et al. and Sharma et al.): contribution of the different Lehmann molecular subtypes in PAM50-defined basal-like **(A)** (n = 75) and non-basal-like **(B)** (n = 17) TNBC. HRD rates (%, Myriad test) associated with each molecular subtype according to Sharma et al. **(C, D)**, (n = 381). In this study, an HRD phenotype was defined by an HRD score >42 according to Myriad. BL1, Basal-like 1, BL2, Basal-like 2, M, Mesenchymal, IM, Immunomodulatory, MSL, Mesenchymal stem like, LAR, Luminal androgen receptor, UNS, unstable, n/d, not documented.

More recent transcriptomic analysis focusing exclusively on TNBC have established that these cancers can also be discriminated into different molecular subtypes. In 2011, Lehmann et al. identified six different subtypes by cluster analysis: Basal-like 1 (BL1), Basal-like 2 (BL2), Immunomodulatory (IM), Mesenchymal (M), Mesenchymal stem-like (MSL) and Luminal androgen receptor (LAR) (64). In this study, the authors showed that deregulated signaling pathways and biological functions were different among subtypes, opening the door to subtype-specific targeted therapies. For example, the BL1 and BL2 subtypes were enriched in cell cycle and DNA damage response genes, the IM subtype in immune response genes and the M and MSL subtypes in EMT and stemness genes (TGF-β, mTOR, Rac1/Rho, Wnt/β-catenin, FGFR, PDGFR, VEGF pathways). Finally, the LAR subtype was enriched in androgen receptor signaling genes. In 2016 and then in 2021 Lehmann et al. simplified their 2011 classification by taking into account the contribution of transcripts from normal immune and stromal cells in the tumor microenvironment. The authors therefore ultimately distinguished 4 subtypes: BL1, BL2, M and LAR (8, 38). Other groups have also performed molecular subtyping of TNBC, leading to the identification of categories that show a relatively high level of concordance with the subtypes originally described by Lehmann et al (9, 10). The relevance of these newer classifications is multifaceted. First, they carry prognostic significance. In the localized setting, Lehmann subtypes have been associated with statistically distinct clinical outcomes. BL1 tumors consistently demonstrate the most favorable prognosis, with higher pathological complete response (pCR) rates following neoadjuvant chemotherapy, as well as improved disease-free survival (DFS) and overall survival (OS). However, findings remain inconsistent across cohorts regarding the prognostic significance of other subtypes, particularly M and BL2 (8, 27, 37–39, 64, 65). For example, in the study by Masuda et al., none of the BL2 cases achieved pCR following anthracycline- and taxane-based neoadjuvant chemotherapy (without platinum agents) (39). In contrast, in the study by Echavarria et al., 47.4% of BL2 tumors achieved a pathological complete response with a carboplatin and docetaxel regimen (37). These findings suggest that tailoring neoadjuvant chemotherapy according to TNBC subtype may be of interest, although this approach requires prospective validation. Second, these classifications may also have therapeutic implications in the advanced setting. Thus, Jiang et al. conducted a phase Ib/II trial evaluating subtype-directed targeted therapies in advanced TNBC, comprising seven treatment arms tailored to molecular subtypes. The overall objective response rate was 29% (66), demonstrating the feasibility of such an approach, although its clinical benefit remains to be confirmed in prospective, randomized studies with larger patient cohorts. Finally, these classifications highlight the molecular heterogeneity within the basal-like subtype defined by the intrinsic classification, at least with respect to the predominant triple-negative component. In fact, Masuda et al. evaluated in their localized TNBC cohort the contribution of each of the Lehmann TNBC molecularsubtypes in basal-like and non-basal-like cancers defined according to the intrinsic PAM50 classification. As shown in **Figure 1**, the basal-like group was mainly made up of the IM (23%), BL1 (21%) and M (20%) subtypes while the non-basal-like group was mainly made up of the LAR (59%) and MSL (35%) ones (39). Interestingly, in the study by Sharma et al., M and BL1 tumors corresponded to the subtypes associated with the highest HRD rates (assessed using Myriad test), at 79% and 76%, respectively. In contrast, MSL and LAR tumors exhibited the lowest HRD rates, at 46% and 24%, respectively (**Figure 1**). Although data for the IM subtype were unavailable and both the IM and MSL subtypes were removed from the second Lehmann classification, these findings suggest that, despite their heterogeneity, basal-like tumors share several fundamental biological features highlighted by the intrinsic classification—namely basal differentiation, EMT, proliferation, and stemness—to which a high level of HRD seems to be frequently associated.

### Factors supporting potential HRD in BLBC: similarities to BRCA1 deficient tumors

BRCA1 is a 24-exon gene located on chromosome 17q21. It acts as a tumor suppressor gene thanks to its various biological functions including cell cycle regulation, cytoskeleton organization, apoptosis regulation, X chromosome inactivation or transcription regulation of certain genes such as TP53 or STAT1 (67, 68). More recently, its role in the differentiation of mammary luminal progenitors has also been reported (59–61, 69–73). Nevertheless, the best-known and most-described function of BRCA1 remains its role in maintaining genome integrity through its involvement in DNA double-strand breaks repair by HR (11). In fact, inactivating germline BRCA1 mutation confers an increased predisposition to certain cancers, in particular ovarian and breast cancer. These breast cancers developed in BRCA1 pathogenic variant carriers are very similar to sporadic BLBC from a clinical, histo-pathological and molecular point of view (**Table 1**). Indeed, from a morphological and histological point of view, BRCA1 mutated tumors and sporadic BLBC are both frequently highly proliferative, with pushing margins, central necrosis, areas of atypical medulary phenotype and frequently peripheral lymphocyte infiltration (14, 58, 63, 74–77). Immunohistochemically, like sporadic BLBC, BRCA1 mutated tumors are largely triple-negative and frequently express basal cytokeratins (CK5/6, CK14) as well as EGFR. A nuclear expression of p53 is also often found. These characteristics similar to BLBC have been extensively studied and reported in particular by Turner et al. (14) and Domagala et al. (77). Interestingly, in addition to these immunohistochemical similarities, Sorlie et al. also showed a high concordance between basal-like and BRCA1-mutated breast cancers in terms of gene expression profile (3). Furthermore, Melchor et al. (78) and Holstege et al. (79) established that chromosomal abnormalities found in BRCA1 mutated tumors are very similar to those found in sporadic BLBC. These similarities concern in particular gains on chromosomes 1p, 2p, 3q, 6p, 6q, 7q, 8q, 10p, 12p, 13q and 19q and losses on chromosomes 3p, 4p, 5q, 10q, 12q, 14q and 15q (79).

**Table 1:** Clinicopathologic and molecular features shared by BRCA1-mutated and sporadic basal-like breast cancers.

	BRCA1m tumors	Sporadic basal-like tumors
Clinical criteria		
Age at onset	Median and mean age: 39-39,9 years (77, 81, 82)	Median age: 45 (51, 52)
Prognosis	Five years OS ~44-91,4% (81, 83, 84)	Five years OS ~60-70% (6, 51, 52)
Histological criteria		
High grade	~66-90% (14, 77, 85)	~70-80% (14, 58)
Proliferation rates	High (14)	High (14, 58)
Borders	Pushing (14)	Pushing (14, 58)
Central necrosis	Present (14)	Present (14, 58)
Areas of medullary or metaplastic phenotype	~38 (14, 77)	~26% (14, 58)
Peripheral lymphocyte infiltration	Present (14)	~56% (14, 58)
Triple negative phenotype	~80% (77)	~77% (4, 5)
EGFR expression	Overexpression 60 – 75% (14, 86)	Overexpression 60 – 80%; amplification 15 – 35% (14, 58)
Basal cytokeratins expression	~65% (14, 86, 87)	~62% (4, 14)
P53 nuclear expression	~58% (86, 87)	High (88)
Chromosomal profile		
Gains/losses	Frequent (78, 79)	Frequent (78, 79)
Gains	1p, 1q, 2p, 3q, 6p, 6q, 7q, 8q, 10p, 13q, 19q (23, 78, 79, 89, 90)	1q, 3q, 6p, 7q, 8p, 8q, 10p, 12p, 17q, 21q (78, 79, 91–94)
Losses	2q, 3p, 4p, 4q, 5q, 8p, 10q, 12q, 13q, 14q, 15q, 17p (23, 78, 79, 89, 90)	1p, 3p, 3q, 4p, 4q, 5q, 8p, 10q, 12q, 13q, 14q, 15q, XP (78, 79, 92–94)

This genomic instability profile common to basal-like and BRCA1 mutated tumors points to an underlying HRD, a process in which the BRCA1 protein is highly involved. This suggests that a part of sporadic BLBC could present BRCA1 inactivation through BRCA1 somatic mutations or epigenetic or post-transcriptional repression of this gene. This hypothesis is supported by frequently reduced BRCA1 transcriptional and protein expression in sporadic BLBC. Thus, Turner et al. (19) compared BRCA1 transcriptional expression in 37 BLBC (identified in IHC by a positive CK5/6 staining) and 37 CK5/6 negative tumors matched on age and grade. BRCA1 mRNA expression was 2-fold lower in BLBC. Additionally, Joosse et al. (23) analyzed by RT-qPCR the mRNA BRCA1 expression level in 41 sporadic BLBC and 83 sporadic luminal tumors, used as control. They showed a reduction in BRCA1 expression in 51% of BLBC. At the protein level, Rakha et al. (80) reported an association between altered BRCA1 IHC nuclear expression and basal-like phenotype defined according to several IHC classifications. For example, 76% of tumors identified as basal-like according to the Nielsen classification (4) showed an alteration in BRCA1 nuclear expression. However, as shown by these different studies, not all BLBC show alterations in BRCA1 transcriptional or protein expression. This suggests that in these tumors, other genes involved in HR could be altered at the transcriptional or post-transcriptional level, also leading to a BRCA-like phenotype called “BRCA-ness” (15–17). In the next sections of this article, we will first review the different techniques that allowed to assess HR alteration in BLBC and secondly, we will examine the identified causes of these alterations.

## Evaluation of HRD in basal-like breast cancers

### Overview of HRD detection methods

HRD can be assessed using several complementary approaches, which can be broadly categorized into four groups: (i) methods based on the detection of chromosomal aberrations, (ii) mutational signature–based approaches, (iii) transcriptomic signatures, and (iv) functional assays evaluating homologous recombination activity in real time. Methods based on chromosomal aberrations rely on the identification of large-scale genomic alterations resulting from defective homologous recombination repair, including loss of heterozygosity (LOH) (95), large-scale state transitions (LST) (96), and telomeric allelic imbalance (TAI) (97). These structural alterations reflect genome-wide instability and can be combined into composite HRD scores (98, 99). An important implementation of this approach is shallow whole-genome sequencing (“shallow HRD”), which uses low-coverage sequencing to infer genome-wide copy number profiles and derive HRD scores (100, 101). This method provides a cost-effective and scalable alternative to high-depth sequencing while maintaining good concordance with established HRD assays. Because these approaches capture cumulative genomic damage acquired during tumor evolution, they primarily reflect historical rather than current HR status. Mutational signature–based approaches identify characteristic patterns of base substitutions, insertions/deletions, and structural rearrangements associated with HRD. Signature 3 is strongly linked to BRCA1/2 alterations, while integrative tools such as HRDetect (102) and CHORD (103) combine multiple genomic features to improve detection sensitivity, particularly in tumors lacking identifiable BRCA mutations. Transcriptomic signatures aim to capture downstream consequences of homologous recombination impairment through gene expression profiling, reflecting alterations in DNA repair pathways, replication stress, and cell cycle regulation (104). Although potentially more dynamic than genomic approaches, their clinical validation remains limited. Functional assays provide a complementary strategy by directly assessing homologous recombination activity in tumor cells. Among these, RAD51 nuclear foci formation following DNA damage is a key surrogate marker of HR proficiency. The absence of RAD51 recruitment indicates functional HRD and has been associated with sensitivity to DNA-damaging agents and PARP inhibitors (105–114). Unlike genomic approaches, functional assays aim to reflect real-time HR capacity, although their implementation remains limited to specialized settings. As summarized in **Table 2**, these methods differ in their technical requirements and level of clinical validation. Comprehensive reviews have detailed the strengths and limitations of these methods (26); therefore, only their relevance to basal-like breast cancers is discussed here.

**Table 2:** Comparative overview of HRD detection methods: cost, turnaround time, availability, clinical validation, and HRD prevalence in BLBC.

Technique	Principle	Estimated cost (EUR/sample)	Turnaround time	Availability	Clinical validation	HRD rate in BLBC
CGH array - MLPA	Detection of CNA	~150–300 €	3–7 days	Limited (research/specialized labs)	Low (lack of standardization)	Not specifically reported
SNP array (LOH, LST, TAI)	Detection of specific chromosomal aberrations	~250–500 €	5–10 days	Moderate (specialized centers)	Good analytical validation	Not specifically reported Good correlation with basal-like intrinsic status
HRD score Myriad myChoice^®^	Composite score (LOH + LST + TAI)	~3,000–4,000 €	10–14 days	Widely available (commercial)	High clinical validation (predictive for PARPi sensitivity in epithelial ovarian tumors)	>75% for BL1 and M Lehmann subtypes
Shallow WGS (shallowHRD)	Low-pass whole genome sequencing (large-scale alterations)	~200–400 €	3–5 days	Emerging (academic + some clinical labs)	Strong analytical validation (≈95% concordance with Myriad)	Not specifically reported
Whole Genome Sequencing (HRDetect, CHORD)	Mutational signatures + rearrangement patterns	~1,000–2,500 €	2–4 weeks	Limited (research/large centers)	Very strong analytical validation	~70–80%
Targeted NGS panels (e.g., SigMA)	Signature 3 inference from targeted sequencing	~300–800 €	5–10 days	Increasing (clinical genomics labs)	Emerging validation	Not specifically reported
Transcriptomic signatures	Gene expression–based HRD inference	~200–600 €	5–10 days	Limited (research/translational platforms)	Moderate validation (Se ~84%, Sp ~73%)	Not specifically reported HRD high cohort enriched in BLBC
RAD51 functional assay	Functional HR activity (RAD51 foci formation)	~100–300 €	1–3 days	Limited (expert centers)	Emerging clinical validation (correlation to olaparib response)	~47% (basal-like PDX)

### Application of HRD detection methods to basal-like breast cancers

Despite the wide availability of HRD detection tools, relatively few studies have specifically evaluated HRD in cohorts strictly defined as basal-like using intrinsic transcriptomic classification. Instead, most available data derive from TNBC cohorts (**Table 3**), in which approximately 70–80% of tumors are basal-like (4, 5, 115), representing an important but imperfect surrogate.

**Table 3:** Evaluation of HRD status in TNBC cohorts.

Trial/reference	Study population	HRD assessment type	% HRD in triple-negative population	Further information
Timms 2014 (98)	All types of breast cancer	HRD score (Myriad)	44.2%	
Isakoff 2015 (116) TBCRC 009	TNBC	HRD LOH HRD LST	43.8% 50%	
Telli 2015 (40) PrECOG	TNBC and/or pathogenic BRCA1/2 mutation, neoadjuvant situation	HRD LOH (positive if ≥ 10) and/or deleterious BRCA1/2 mutation. HRD LOH test on pre-treatment biopsies.	76.9%	78% of TNBC were identified as basal-like according to the intrinsic classification. According to Lehmann classification: 16% BL1, 4% BL2, 27% IM, 8% LAR, 27% M, 6% MSL, 12% unstable
Handled 2016 (20)	TNBC	HRD LST	52%	
All 2018 (48) DTT	Unselected advanced TNBC	HRD score (Myriad) (positive if ≥ 42) and/or tumor pathogen BRCA1/2 mutation	41.5%	
Law 2018 (43) GEPARSIXTO	Stage II-III TNBC, neoadjuvant situation	HRD score (Myriad) (positive if ≥ 42) and/or tumor pathogen BRCA1/2 mutation	70.5%	60.3% HRD+ without BRCA1/2 mutation
Sharma 2018 (27) SWOGS9313	TNBC, adjuvant situation	HRD score (Myriad) (positive if ≥ 42) and/or tumor pathogen BRCA1/2 mutation	67%	
Mayer 2020 (42) TBCRC030	Stage II-III TNBC, neoadjuvant situation.	HRD score (Myriad) (positive if ≥ 33)	71.1%	

#### Chromosomal aberrations-based approaches

Chromosomal instability–based HRD scores consistently report high rates of HRD in TNBC populations (**Table 3**). In the neoadjuvant setting, Telli et al. reported HRD positivity in up to 76.9% of TNBC cases using LOH-based criteria (40). Sharma et al. further refined this observation by analyzing HRD across TNBC molecular subtypes (27). They reported overall HRD rates of approximately 67%, with marked enrichment in basal-like–related subtypes, reaching 76% in BL1 tumors and 79% in M tumors. In contrast, LAR and MSL subtypes exhibited substantially lower HRD rates. These findings are supported by the independent analysis of Imanishi et al. (28), who also evaluated HRD across TNBC molecular subtypes using chromosomal aberration–based scores. In this study, BL1 and M subtypes again displayed the highest HRD rates (79% and 76%, respectively), whereas LAR and MSL tumors showed lower rates (24% and 46%). Together, these studies consistently indicate that HRD-related genomic alterations are enriched within basal-like–associated subtypes, while highlighting persistent heterogeneity across TNBC. This approach was further explored in patient-derived xenograft (PDX) models by Ter Brugge et al. (29), using shallow whole-genome sequencing to derive genomic HRD scores in a cohort of 55 TNBC PDX. In this study, approximately 50% of the models were classified as HRD-positive using this approach. Although intrinsic PAM50 classification was not systematically reported for the entire cohort, molecular subtyping using the SCMGENE model—a reduced gene expression–based surrogate of the PAM50 classifier based on a limited set of informative genes—indicates that the great majority of these models exhibit a basal-like profile. This suggests that the cohort represents a basal-like–enriched population, thereby strengthening the relevance of these findings for BLBC.

#### Mutational signature-based approaches

Whole-genome sequencing studies have demonstrated enrichment of HRD-associated mutational signatures in basal-like tumors. Nik-Zainal et al. identified a strong association between COSMIC signature 3 and basal-like phenotype, particularly in tumors with BRCA1 alterations (30). Similarly, Polak et al. showed that RAD51C promoter methylation is associated with signature 3 and occurs preferentially in basal-like contexts (31). More integrative approaches such as HRDetect have further refined HRD detection. Hohmann et al. studied 184 TNBC cases classified as basal-like according to the PAM50 classification. Among these cases, 71% were classified as HRD-positive by HRDetect (117). Chopra et al. reported that a large majority (80%) of basal-like tumors within TNBC cohorts were classified as HRDetect-high, whereas non-basal tumors were less frequently positive (32). Staaf et al. similarly observed enrichment of HRDetect-high cases in basal-like tumors, although a significant subset remained HRDetect-low, underscoring intra-subtype heterogeneity (33).

#### Transcriptomic signatures

Transcriptomic approaches provide complementary insight into HRD by capturing downstream transcriptional consequences of DNA repair impairment. Walens et al. demonstrated that HRD-associated gene expression profiles are enriched in basal-like tumors and associated with poorer prognosis (104). In the I-SPY2 trial, Severson et al. identified HRD-positive tumors in approximately 78% of TNBC using a transcriptomic signature, with improved response to combined PARP inhibitor and platinum-based therapy (22). However, these approaches remain insufficiently validated in strictly defined BLBC cohorts.

#### Functional test: RAD51 nuclear recruitment

Functional evaluation of homologous recombination through RAD51 nuclear foci formation provides a direct assessment of DNA repair capacity and represents a key complement to genomic approaches. In basal-like cell line models, RAD51-based assays have already highlighted substantial heterogeneity, with only a subset of cell lines showing impaired RAD51 recruitment, while others retain homologous recombination proficiency despite basal-like features (24). In the study by Ter Brugge et al., functional HR capacity was assessed in a subset of 32 TNBC PDX models. Importantly, all these models clustered within the basal-like group based on the SCMGENE surrogate of PAM50 classification, indicating that functional analyses were performed in a molecularly homogeneous basal-like population (29). Despite this, only 46.9% of models were classified as HR-deficient based on RAD51 foci formation, while 53.1% retained homologous recombination proficiency. This discordance between genomic HRD scores and functional RAD51 status highlights important biological considerations. Genomic scar–based approaches capture historical HRD and may remain positive even after restoration of homologous recombination function. Secondary reversion events in homologous recombination genes, particularly BRCA1/2, can restore DNA repair capacity while leaving persistent genomic scars. In addition, intratumoral heterogeneity and clonal evolution under treatment pressure may lead to the coexistence of HR-deficient and HR-proficient cell populations. Differences in assay sensitivity, thresholds, and technical implementation may also contribute to this discordance. Collectively, these mechanisms help explain why genomic HRD scores may overestimate the proportion of tumors with true functional HRD.

Taken together, available data indicate that HRD-related features are enriched in basal-like breast cancers across multiple methodological approaches, including chromosomal aberration–based scores, mutational signatures, and transcriptomic analyses. However, this enrichment is not universal and varies depending on the detection method used. Importantly, functional assays consistently indicate that only a subset of basal-like tumors exhibit true homologous recombination deficiency. This distinction highlights the biological heterogeneity within BLBC and suggests that genomic HRD markers alone may overestimate the proportion of tumors with clinically relevant HRD. These observations support a model in which basal-like breast cancers are enriched for HRD-related alterations but encompass both HR-deficient and HR-proficient tumors. Integrating intrinsic subtype classification with functional HRD assessment may therefore represent a more accurate strategy to define clinically actionable HRD in this context.

## HRD causes in basal-like breast cancer

Beyond BRCA1 and BRCA2 which constitute important predisposition genes, many other genes are involved in HR (11). Some of them may be the target of different inactivation mechanisms: epigenetic repression (for example by promoter hypermethylation), inactivating mutation, post transcriptional silencing (118). Generally, in HRD tumors, the 2 alleles are found inactivated, although several studies showed that mono-allelic BRCA1 inactivation (haplo-insufficiency) is already associated with a certain level of genetic instability that could favor the subsequent appearance of an HRD phenotype (119–122). In this chapter we will therefore detail the different HR genes inactivation mechanisms involved in the acquisition of an HRD phenotype in BLBC (see also **Table 4**).

**Table 4:** Main suspected HRD causes in BLBC.

Category	Mechanism	Gene(s)/Process	Frequency in BLBC	Key Evidence/Notes
Genetic alterations	Germline mutations	BRCA1	~10% (TCGA)	Strong association with basal-like phenotype
		BRCA2	~2% (TCGA)	
	Somatic alterations	BRCA1	~5% (TCGA)	
		BRCA2	~5% (TCGA)	
	Other HR gene mutations	RAD51C, ATM, CHEK1, RAD51, WRN, PALB2, BARD1	<1-2%each ~6% collectively (TCGA)	
Epigenetic/transcriptional	Promoter hypermethylation	BRCA1	~14 – 34%	Impact depends on methylation level (≥90% → full silencing)
	Other methylation events	RAD51C, FANCF	Rare	Limited role in BLBC
	Transcriptionnal repression	ID4 overexpression	~40–50% high expression	Strongly associated with basal-like phenotype
	Trancriptional regulators	EMSY amplification	Rare in BLBC	BRCA2 repression More frequent in luminal B tumors
		HOXA9 loss	Unclear in BLBC	May reduce BRCA1 expression Limited evidence
		HMGA1b overexpression	Very rare	BRCA1 repression Limited evidence
Post-transcriptional	miRNA-mediated repression	miR-146a/b, miR-182, miR-17, miR-26a, miR-638, miR-1245	Not precisely quantified in BLBC	BRCA1/2 downregulation Mainly preclinical data

### Genetic alterations

**Figure 2:**
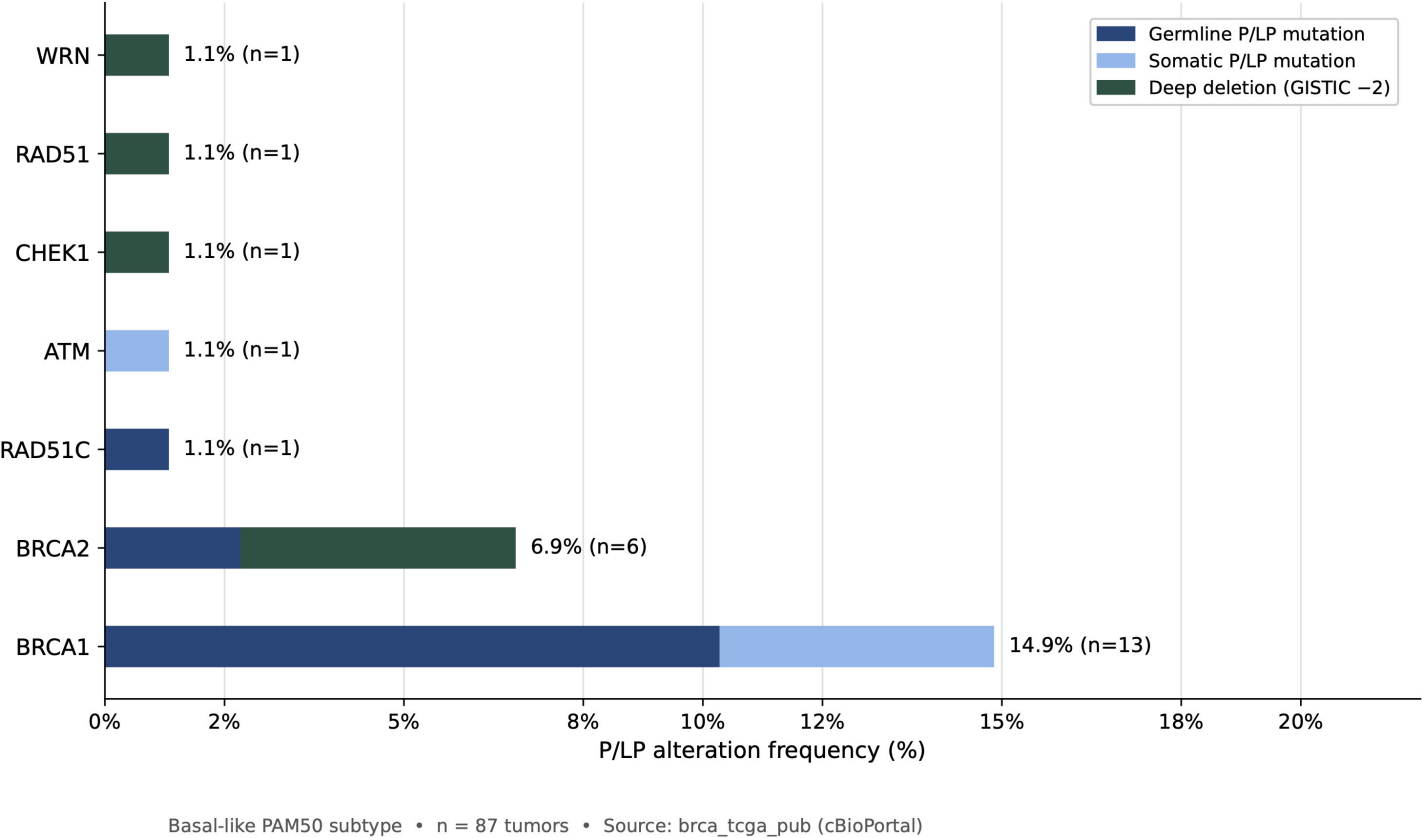
Frequency of pathogenic/likely pathogenic alterations in homologous recombination genes in basal-like breast cancer (TCGA BRCA). Dark blue: germline pathogenic/likely pathogenic (P/LP) mutations, as annotated in the cBioPortal dataset. Light blue: somatic P/LP mutations. Dark green: homozygous deep deletions (GISTIC -2 score). Only genes harboring at least one P/LP alteration are shown; genes with exclusively variants of uncertain significance (VUS) or no alteration are omitted. Percentages indicate the fraction of basal-like tumors carrying each alteration type; n indicates the absolute number of altered samples out of 87. P/LP mutations include: loss-of-function variants (nonsense, frameshift, splice-site) classified as Pathogenic by ACMG rules, and missense variants with established ClinVar P/LP annotation (BRCA1 C61G and R1699W). VUS were excluded (BRCA2 E2650Q, C3304S, R2336C, N3124I; ATM N796T; BRCA1 G1788V; PTEN S10R; CHEK2 D203E; FANCD2 A731V; BRIP1 L717V).

Basal-like breast cancers are characterized by a relatively high frequency of alterations affecting homologous recombination genes, with BRCA1 being the most prominently involved gene. Germline *BRCA1* mutations are detected in a substantial fraction of TNBC, with reported frequencies ranging from approximately 6.5% to 34.4% in selected cohorts (123, 124) and 6.5% to 18% in unselected populations (125–131). Although estimates specifically restricted to BLBC remain limited, data from *TCGA* indicate that *BRCA1* mutations account for 14.9% of cases, including 10.3% germline and 4.6% somatic mutation (18). In contrast, germline BRCA2 mutations are less frequent, occurring in approximately 0–9% of selected TNBC cohorts (123, 124)and 1-4% of unselected TNBC populations (125–131). In BLBC specifically, TCGA data report BRCA2 alteration in 6,9% of cases comprising 2,3% germline and 4,6% somatic deep deletions (18). According to TCGA, alterations in other HR-related genes such as WRN, RAD51, CHEK1, ATM, RAD51C (**Figure 2**) are uncommon in basal-like tumors, each typically occurring at frequencies below 1–2%, and collectively accounting for only a limited proportion of HR-deficient cases (18, 132). In addition to TCGA findings, alterations involving other homologous recombination genes, including PALB2 and BARD1, have also been described, although these observations were essentially made in TNBC cohorts and not in specifically basal-like tumors (31, 118, 127, 129, 133, 134). This low mutational burden in canonical HR genes further supports the notion that non-genetic mechanisms could contribute substantially to HRD in BLBC.

### Transcriptional dysregulations

Even if the number of genes potentially involved in the acquisition of an HRD phenotype is substantial, mutations and CNA relating to these genes do not on their own explain all the cases of HRD in BLBC/TNBC. It has therefore been proposed that transcriptional deregulation of some of these genes could constitute alternative phenomena explaining HRD in these cancers.

#### BRCA1 promoter hypermethylation

Early studies investigating BRCA1 promoter hypermethylation in basal-like breast cancer have reported heterogeneous results. In the study by Matros et al. (2005), BRCA1 promoter hypermethylation was observed in 21% (16/75) of sporadic breast cancers, but appeared to be less frequent in basal-like tumors (135). These findings were supported by Turner et al. (2007), who analyzed 37 sporadic breast cancers expressing basal cytokeratins (CK5/6) alongside matched controls. In this cohort, BRCA1 promoter hypermethylation was detected in 14% of basal-like tumors compared to 11% in controls, with no significant difference (p = 0.72) (19). In contrast, Joosse et al. reported higher rates of BRCA1 promoter hypermethylation in a cohort of 41 basal-like breast cancers, where it was identified in 34% (14/41) of cases and significantly associated with reduced BRCA1 transcriptional expression (23). Similarly, in a study by Bal et al., BRCA1 promoter hypermethylation was found in 24% (11/45) of sporadic breast cancers, all of which exhibited reduced or absent BRCA1 protein expression and a basal-like phenotype (136). Beyond prevalence, the clinical implications of BRCA1 promoter methylation remain complex. In the PETREMAC phase II trial, conducted in patients with stage II–III triple-negative breast cancer, BRCA1 promoter hypermethylation was associated with improved response to olaparib, suggesting a functional link with homologous recombination deficiency (107). However, contrasting results were reported by Ter Brugge et al. in a study of 55 patient-derived xenograft (PDX) models of early TNBC. In this setting, BRCA1 promoter methylation was not consistently associated with increased sensitivity to platinum-based chemotherapy. Notably, only high levels of methylation (90–100%) were linked to complete BRCA1 transcriptional silencing, whereas intermediate levels (40–60%) were associated with residual BRCA1 expression and therapeutic resistance (29). Taken together, these findings suggest that while BRCA1 promoter hypermethylation is present in a subset of basal-like breast cancers, its frequency is variable across studies and its functional and clinical impact seems to depend on the extent of methylation, rather than its mere presence.

#### Id4 overexpression

The Id family proteins (Id1–Id4) are proteins possessing a helix-double-helix dimerization domain thanks to which they can form heterodimers with transcriptional factors also structured in a basic helix-loop-helix. As the Id proteins do not have a DNA-binding domain, the heterodimers that they form with the transcription factors antagonize the latter by preventing them from interacting with the DNA. They are therefore considered as dominant negative transcriptional regulators (137). In the Id family, Id4 has notably been identified as a negative regulator of BRCA1 expression (138), notably in certain sporadic Erα negative breast cancers (139). Moreover, in the study by Branham et al., Id4 demethylation was positively correlated with its transcriptional and protein expression and strongly associated with a BRCAness phenotype measured by MLPA in 63 breast tumors (140). Interestingly, Id4 overexpression is strongly associated with the basal-like phenotype.

Thus, in the study by Turner et al., Id4 expression level (evaluated by qPCR) in the 37 sporadic BLBC studied was 9.1 times higher than in the non-basal-like controls matched on age and grade (p < 0.0001) (19). Additionally, Wen et al. compared the Id4 protein expression level in IHC in 101 TNBC compared with 113 non-TNBC. In this study, 75% (76/101) of the TNBC cases showed immunoreactivity for Id4, including 40% (40/101) with positive staining in more than 50% of the tumor cells. Conversely, only 5% (6/113) of control cases were positive for Id4 (p < 0.0001). Id4 expression was significantly associated with nuclear grade, mitotic index and basal cytokeratin CK14 expression (not significant for CK5/6 and EGFR) (141). Similarly, Thike et al. evaluated Id4 status in IHC in 699 TNBC cases organized in TMA. In this study, Id4 expression was found in 95% (663/699) of the cases among which 45.6% (317/699) of intense expression and 65% (454/699) of expression in more than 50% of tumor cells. Id4 expression was significantly associated with stage, grade, mitotic index and basal-like phenotype defined in IHC by CK14, EGFR and 34βE12 expression (142). Additionally, Junankar et al. analyzed Id4 protein expression level in IHC in a cohort of 80 BLBC. In this study, 37.5% (30/80) of the cases had a high level of Id4 expression (Id4 high). Id4 high status was significantly associated with an unfavorable prognosis in terms of overall survival at 3 years (hazard ratio for death: 4.24, p<0.0008), independently of grade or proliferation estimated by Ki67. The same unfavorable prognostic impact was found for Id4 transcriptional expression in 2 other BLBC independent cohorts of 60 and 285 cases respectively. In these cohorts, Id4 was highly expressed in approximately 50% of cases. It should be noted that in this same work, Id4 repression in BLBC cell models was associated *in vitro* with less proliferation and *in vivo* with less tumor growth, which could explain the difference in prognosis between Id4 high and Id4 low cohorts (21). It should also be noted that in TCGA, ID4 high BLBC trancriptome was enriched in numerous genes involved in mammary stem cells’ biology. This relationship between Id4 and stemness could also partly explain the negative prognostic impact of Id4 (21). Nevertheless, the Id4 biological functions specifically in BLBC remain obscure. Therefore, Baker et al. analyzed by Chip-seq the Id4-chromatine interactome in different BLBC and high-grade ovarian serous carcinomas cell lines. They also identified by Rapid immunoprecipitation and mass spectrometry of endogenous proteins (RIME) all of Id4 protein interactions in these models. Interestingly, they showed that in addition to its canonical role as a dominant negative transcriptional regulator, Id4, in BLBC, could interact with certain areas of euchromatin, even in the absence of any known DNA binding domain. These interaction zones correspond to sites of active transcription and DNA damage. At this level, Id4 does not seem to influence transcription but rather interact with many proteins involved in DNA damage response. The exact consequences of these interactions on genome integrity maintenance and genomic stability remain to be determined, but these discoveries could make the link between Id4 overexpression and BRCAness in certain BLBC (143).

#### Other mechanisms

Several other mechanisms of transcriptional and epigenetic deregulation of HR actors have been described in mammary carcinomas and recently reviewed by Mekonnen et al (144). These mechanisms notably include RAD51C and FANCF hypermethylation. Thus, in the study by Polak et al. RAD51C promoter hypermethylation was well correlated with a significant drop in RAD51C mRNA expression and strongly associated with signature 3. Interestingly, among patients with this epigenetic abnormality, there was an enrichment in young Afro-descendants with basal-like cancer (31). Nevertheless, the contribution of RAD51C promoter hypermethylation in the acquisition of an HRD phenotype in TNBC/BLBC seems weak. In fact, in the study by Manié et al., only 4% (3/69) of cases in HRD+ TNBC (LST+) presented this alteration (20).

Similarly, although Turner et al. originally proposed FANCF promoter hypermethylation as an HRD mechanism (16), the frequency of this alteration is less than 1% in the Wei et al. multi-subtype breast cancer cohort (145).

Furthermore, EMSY is a gene located on chromosome 11q13 whose protein interacts with exon 3 of BRCA2 gene leading to its transcriptional repression (146). EMSY overexpression *in vitro* leads to the accumulation of chromosomal aberrations similar to those observed in the context of BRCA2 inactivating mutation, making amplification/overexpression of EMSY a potential cause of HRD (147). EMSY amplification has been described in a number of sporadic breast cancers and is correlated with poor prognosis (146–153). Nevertheless, this alteration is rare in BLBC and is rather associated with the luminal B phenotype (147, 151).

Homeobox (HOX) family genes are morphogens encoding transcription factors regulating the expression of many genes involved in cell growth and differentiation. They are frequently dysregulated in many cancers (154). Gilbert et al. thus identified HOXA9 as a positive regulator of BRCA1 expression and showed that its mRNA level was significantly lower in their cohort of 47 TNBC, compared to non-tumor controls (155). Nevertheless, the consequences of HOXA9 loss on HRD phenotype acquisition remain to be determined. Moreover, in TCGA mammary cohort, HOXA9 does not appear to be inactivated or repressed in BLBC (18).

Finally, HMGA1b is a protein able to bind to DNA in AT-rich region and interact with various transcription factors in order to modulate their activity. Baldassarre et al. demonstrated that this protein could interact directly with BRCA1 promoter leading to its transcriptional repression (156). However, HMGA1b overexpression remains relatively rare in BLBC (only 1 amplification case in TCGA mammary cohort (18)) and the consequences in terms of HRD also remain to be determined.

### Post-transcriptional dysregulations

In addition to the previously detailed HRD mechanisms, some teams sought to explore the impact of certain miRNAs on the post-transcriptional regulation of certain HR actors, in particular BRCA1. Thus Garcia et al. demonstrated the ability of miR-146a and miR-146b-5p to repress BRCA1. As expected, in this study, the highest expression levels of these miRNAs were found in BLBC cell lines and in TNBC tissues (157). By studying TCGA data, M’hamed et al. confirmed that miR-146a is significantly overexpressed in TNBC compared to non-TNBC. However, they failed to demonstrate the ability of this miRNA to repress BRCA1 in the MDA-MB-231 mesenchymal-like TNBC cell line, unlike miR-10b and miR-26a (158). Additionally, Shen et al. and Pastrello et al. showed that certain miR-26a polymorphisms were associated with an earlier breast cancer diagnosis in certain risk groups, highlighting the hypothetical impact of this miRNA in the tumorigenesis of certain breast cancers (159, 160).

Furthermore, Moskwa et al. demonstrated a statistically significant inverse correlation between expression level of another miRNA, miR-182, and BRCA1 in a panel of TNBC cell lines. Conversely, they also showed that in these models, miR-182 repression led to BRCA1 overexpression, suggesting a role for this miRNA in the post-transcriptional regulation of BRCA1 (161, 162).

Shen et al. and De Summa et al. showed that another miRNA, miR-17, was able to interact with BRCA1 3’UTR mRNA and inactivate it (163, 164). This miRNA was found overexpressed in the MDA-MB-231 cell line (164).

Additionally, Tan et al. also identified BRCA1 transcript as a direct target of miR-638 in TNBC cell lines. In their study, overexpression of this miRNA induced hypersensitivity to Cisplatin. Nevertheless, it was found rather under-expressed in the triple-negative primary tissues of their cohort (165).

Additionally, Quan et al. showed that several miRNAs of the miR15/107 family are able to interact directly with the coding sequence of BRCA1 mRNA, inducing its inactivation. Nevertheless, the consequences in terms of protein expression were heterogeneous in the pancreatic and colorectal cancer cell lines used (166). Moreover, these miRNAs seem rather under-expressed in the context of mammary carcinomas (167).

Finally, Song et al. demonstrated in the MDA-MB-231 cell line that miR-1245 is capable of suppressing BRCA2 transcript activity via interaction with its 3’ UTR region, resulting in the appearance of HRD, genomic instability and increased sensitivity to irradiation (168). Nevertheless, the abundance of miRNAs specifically in BLBC and its impact on HR status *in vivo* remains to be determined.

## Therapeutic implications

Given the HRD frequency in BLBC, they should theoretically be relatively sensitive to platinum salts and PARPi, as is particularly the case in epithelial ovarian cancers (34–36). In most studies, the analysis of response to these agents and the impact of their use on survival focused on cohorts of TNBC, as they represent approximately 80% of BLBC practice. **Table 5** therefore lists the main trials in these situations.

**Table 5:** Main studies reporting the sensitivity of triple-negative breast cancers to platinum salts and PARP inhibitors.

Trial/reference	Population	Stage	Analysis sub-groups according to HR status	Treatments tested	Judgment criteria	Results
PETREMAC/Eikesdal (107) Phase II single arm	Unselected TNBC (Neo-adjuvant) 32 patients	II/III		olaparib 300mg x2/d for a maximum of 10 weeks	-main: objective response rate (ORR)	-ORR = 56.3% (18/32) -88.9% (16/18) of responders vs 28.6% (4/14) of non-responders had HR gene mutations or BRCA1 hypermethylation (p = 0.0008)
PrECOG 0105/Telli (40) Stage II	Unselected TNBC (Neo-adjuvant) 80 patients	I to IIIA	A: HRD-LOH high (n = 50) B: no HRD-LOH low (n = 15)	gemcitabine (1000 mg/m2 D1 D8) + carboplatin (AUC 2 D1 D8) + iniparib (5.6 mg/kg D1 D4 D8 D11) every 21 days x 6 cycles	-main: pathological complete response (pCR) and residual cancer burden (RCB) 0/1	- overall pCR = 36% A: RCB 0/1: 66% B: RCB 0/1: 20%
SOLTI NeoPARP/Llombart-Cussac 2015 (169) Stage II	Unselected TNBC (Neo-adjuvant) 141 patients	II/IIIA		paclitaxel 80 mg/m2 weekly (P) vs paclitaxel 80 mg/m2 weekly + iniparib 11.2 mg/kg weekly (PI1) vs paclitaxel 80 mg/m2 weekly + iniparib 5.6 mg/kg twice weekly D1 D4 (PI2)	-main: pCR	-pCR P: 21% -pCR PI1: 22% -pCR I2: 19%
GeparOLA/Fasching (170) Phase II	HRD (Myriad) or BRCA1/2m breast cancers 107 patients	I to III		paclitaxel 80 mg/m2 weekly + olaparib 100 mg x2/day (PO) vs paclitaxel 80 mg/m2 + carboplatin AUC twice weekly (PC) 12 weeks After the 12 weeks, 3 to 4 cycles of epirubicin + cyclophosphamide	-main: pCR	-pCR PO 55.1%, 90% CI 44.5% to 65.3% vs pCR PC 48.6%, 90% CI 34.3% to 63.2%)
Fountain (171) Phase II single arm	Unselected TNBC (Neo-adjuvant) 63 patients	II/III		paclitaxel 80 mg/m2 weekly + carboplatin AUC 2 weekly for 12 weeks then epirubicin/cyclophosphamide dose dense 4 cycles	-main: pCR	-pCR: 54%
Sharma (27) Phase II single arm	Unselected TNBC (Neo-adjuvant) 190 patients	I/III	A: BRCAm B: BRCA wild type	carboplatin AUC 6 + taxotere 75mg/m2/3 weeks 6 cycles	-main: pCR -secondary: RCB0/1	-pCR: 55% pCR A 59% pCRB 56% (p=0.83) -RCB0/1: 68%
I-SPY-2/Rugo (172) Phase II	TNBC (Neo-adjuvant) 72 patients	II/III		paclitaxel 80 mg/m2 weekly + veliparib 50 mg x2/day + carboplatin AUC6/3 weeks, 12 weeks (PVC) Vs paclitaxel alone (P) After 12 weeks 4 cycles of doxorubicin/cyclophosphamide in both arms	-main: pCR	-pCR PVC 51% (95% Bayesian probability interval, 36 to 66%) vs pCR P 26% (95% PI, 9 to 43%)
Litton (173) Phase II	HER2- breast cancers with gBRCA1/2m (75% triple-negative) (Neo-adjuvant) 20 patients	I to III		talazoparib 1 mg/day 6 months	-main: RCB0/1	-RCB0: 53% -RCB0/1: 63%
Olympia/Tutt (174) Phase III	HER2- breast cancers with gBRCA1/2m (> 80% triple-negative) and at high clinicopathologic risk of recurrence (Adjuvant) 1836 patients	I to III		olaparib 300 mg x2/d vs placebo, 52 weeks	-main: invasive disease-free survival (iDFS) -secondary: overall survival (OS)	-iDFS at 4 years olaparib 82.7% vs placebo 75.4% (HR: 0.63, 95% CI 0.50 – 0.78) - OS at 4 years olaparib 89.8% vs placebo 86.4% (HR: 0.68, 95% CI 0.47 – 0.97)
Silver (175) Phase II single arm	Unselected TNBC (Neo-adjuvant) 28 patients	II/III		cisplatin 75 mg/m2/3 weeks 4 cycles	-main: pCR -secondary: ORR -identification of predictive biomarkers of response	-pCR: 22% (6/28) including the 2 gBRCA1m -ORR: 64% (18/28) All cases were classified as basal-like according to the intrinsic classification. Factors associated with a good response to Cisplatin: young age (p = 0.001), low BRCA1 mRNA expression (p = 0.03), BRCA1 promoter methylation (p = 0.04), p53 inactivating mutation (p=0.01), E2F3 activation signature (p=0.03).
Yuan (176) Phase II single arm	Unselected TNBC (Neo-adjuvant) 67 patients	II/III		carboplatin AUC6/4 weeks 4 cycles + nab-Paclitaxel 100 mg/m2 weekly 16 weeks	-main: pCR/RCB	-pCR (RCB0): 48% (32/67) -RCB1: 15% (10/67) -RCB2: 28% (19/67) -RB3: 7% (5/67) -progression: 2% (1/67)
NeoSTOP/Sharma (177) Stage II	Unselected TNBC (Neo-adjuvant) 100 patients	I to III		carboplatin AUC 6/3 weeks 4 cycles + paclitaxel 80 mg/m2 weekly 12 weeks then doxorubicin + cyclophosphamide/2 weeks 4 cycles (CPDC) Vs carboplatin AUC6/3 weeks + taxotere (CT) 75mg/m2/3 weeks 6 cycles	-main: pCR/RCB -secondary: Relapse Free Survival (RFS) and Overall survival (OS)	-pCR CPDC 54% (95% CI:40%-69%) vs CT 54% (95% CI: 40%-68%). -RCB 0+I: 67% in both arms. No difference in RFS and OS.
TBCRC031/Tung (178) Phase II	Localized breast cancers with gBRCAm (70% triple-negative cancers) (Neo-adjuvant) 118 patients	I to III		cisplatin 75 mg/m2/3 weeks 4 cycles vs doxorubicin 60mg/m2 + cyclophosphamide 600mg/m2/2 to 3 weeks 4 cycles (AC)	-main: pCR -secondary: probability RCB0/1	-pCR cisplatin 18% Vs pCR AC26% (RR: 0.70, 95% CI 0.39 – 1.2) -probability RCB0/1 cisplatin 33% vs AC 46% (RR 0.73, 95% CI 0.50 – 1.1)
Byrsky (179) Phase II	Localized breast cancers with BRCA1m (76.6% triple-negative) (Neo-adjuvant) 107 patients	I to III		cisplatin 75 mg/m2/3 weeks 4 cycles	-main: pCR	-PCR: 61% (65/107)
WSG-ADAPT-TN/Gluz (180) Phase II	Unselected TNBC (Neo-adjuvant) 336 patients	I to III		nab-Paclitaxel 125 mg/m2 + gemcitabine 1g/m2 D1 D8/3 weeks (NG) vs nab-Paclitaxel 125mg/m2 + carboplatin AUC2 D1 D8/3 weeks AUC2 (NC) 12 weeks	-main: pCR	-pCRNG 28.7%, 95% CI: 0.22 to 0.36 vs NC 45.9%, 95% CI: 0.38 to 0.54; P = 0.002
Brightness/Loibl (181) Phase III	Unselected TNBC (Neo-adjuvant) 634 patients	II/III		paclitaxel (80 mg/m² weekly for 12 weeks) + carboplatin (AUC 6/3 weeks 4 cycles) + veliparib (50 mg x2/day) (PCV) vs paclitaxel (80 mg/m² weekly 12 weeks) + carboplatin (AUC 6/3 weeks 4 cycles) + pacebo (PC) vs paclitaxel (80 mg/m² weekly for 12 weeks) alone (P) Followed by 4 cycles of doxorubicin/cyclophosphamide in the 3 arms	-main: pCR -secondary: RFS and OS	-pCR PCV vs P: 53% (168/316) vs 31% (49/158), p<0 0001 -pCR PCV vs PC: 53% (168/316) vs 58% (92/160), p = 0.36 -RFS: HR PCV vs P=0.63, 95% CI: 0.43-0.92, *P* =0.02 HR PCV vs PC = 1.12, 95% CI: 0.72-1.72, *P* = 0.62) -OS: no significant difference between the groups
Kaklamani (41) Phase II single arm	Unselected TNBC (Neo-adjuvant) 30 patients	I/III	A: HRD(Myriad) and/or BRCAm B: no HRD	carboplatin AUC 6/3 weeks + eribulin 1.4 mg/m2 D1-D8 4 cycles of 21 days	-main: pCR	-pCR: 43% (13/30) -PCR group A: 75% (9/12) -pCR group B: 14.2% (2/14)
TBCRC30/Mayer (42) Phase II	Unselected TNBC (Neo-adjuvant) 48 patients	I to III	A: HRD (Myriad) or tBRCAm B: HRP	cisplatin 75 mg/m2/3 weeks (n=56) vs paclitaxel 80mg/m2 weekly 12 weeks	-main: RCB0/1 vs RCB2/3	-RCB0/1 cisplatin = 26.4% vs RCB0/1 paclitaxel = 26.3% (ns) A: RCB0/1: 23% vs. 12%; OR 2.22 (95% CI: 0.39–23.68); ns B: RCB0/1: 29% vs. 31%; OR 0.90 (95% CI: 0.19–4.95); ns
CALGB40603/Sikov (182) Phase III	Unselected TNBC (Neo-adjuvant) 443 patients	II/III		paclitaxel 80 mg/m2 weekly 12 weeks vs paclitaxel 80 mg/m2 weekly 12 weeks + bevacizumab 10 mg/kg/2 weeks 9 cycles vs paclitaxel 80 mg/m2 weekly 12 weeks + carboplatin AUC6/3 weeks 4 cycles vs Paclitaxel 80 mg/m2 weekly 12 weeks + carboplatin AUC6/3 weeks 4 cycles + bevacizumab 10 mg/kg/2 weeks 9 cycles followed by doxorubicin/cyclophosphamide 4 cycles after the first 12 weeks of treatment in each arm	-main: pCR -secondary: RFS and OS	-pCR carboplatin groups 54% vs other groups 41%; P.0029 -no significant differences in RFS and OS
GEPARSIXTO/Loibl (43) Phase III	Unselected TNBC (Neo-adjuvant) 595 patients	II/III	2 subgroups A: HRD (Myriad) and/or tBRCAm B: HRP	paclitaxel 80 mg/m2 weekly + non-pegylated liposomal doxorubicin 20 mg/m2 weekly + bevacizumab 15 mg/kg/3 weeks (n = 157) 18 weeks (PDB) vs same treatment + carboplatin AUC 2 weekly 18 weeks (PDBC)	-main: pCR -secondary: RFS and OS	-pCR PDB: 36.9% (84/158) vs PCR PDBC: 53.2% (58/157), p=0.005. A: pCR 33.9% vs. 63.5%; GOLD 3.4 (95% CI: 1.7–6.9) B: pCR 20.0% vs. 29.6%; GOLD 1.7 (95% CI: 0.5–5.7) - improvement in RFS in the PDBC group (hazard ratio 0.56 (95% CI 0.34–0.93); P = 0.022). A: HR 0.49 (95% CI: 0.23–1.04); P = 0.059 B: HR 0.44 (95% CI: 0.17–1.17); P = 0.086) -no significant difference in OS
TBCRC048/Tung (45) Phase II	Metastatic breast cancers with alterations of genes involved in homologous recombination (19% of triple-negative breast cancers) Pre-treatment with a maximum of 2 lines of chemotherapy in the metastatic phase, no previous treatment with PARPi or progression on platinum salts. 27 patients in each cohort	IV	- Cohort 1: ATM, ATR, BAP1, BARD1, BLM, BRIP1, CHEK1, CHEK2, CDK12, FANCA, FANCC, FANCD2, FANCF, MRE11A, NBN, PALB2, RAD50, RAD51C, RAD51D, Or WRN germline mutation -Cohort 2: somatic mutation in one of the same genes or BRCA1/2	olaparib 300mg x 2/day	-main: ORR	-Cohort 1: ORR 33% (9/27); the 9 cases with gPALB2m ORR in the gPALB2m population: 82% (9/11) -Cohort 2: ORR 31% (8/26) ORR non BRCA1/2mutated population: 0% (0/10)
Gelmon (183) Phase II single arm	Metastatic TNBC or with BRCA1/2m (81% triple-negative) 26 patients	IV		olaparib 400 mg x2/day	-main: ORR	-ORR: 0%
Kummar (184) Stage II	TNBC not selected (2^nd^ metastatic line or more) 21 patients	IV		cyclophosphamide 50 mg x1/day (n=18) Vs cyclophosphamide 50 mg x1/day + veliparib 60 mg/day	-main: ORR -secondary: progression-free survival (PFS)	-ORR cyclophosphamide = 5.6% (1/18) vs ORR cyclophosphamide + veliparib = 9.5% (2/21), ns -PFS cyclophosphamide = 1.9 months vs PFS cyclophosphamide + veliparib = 2.1 months, ns.
Byrsky (185) Phase II	Metastatic breast cancers with BRCA1m (70% triple-negative) Possibility of having received up to 4 lines of prior chemotherapy in the metastatic phase. 20 patients	IV		cisplatin 75mg/m2/3 weeks 6 cycles	-Main: ORR	-ORR: 80% (complete response 45% (9/20) and partial response 35% (7/20))
SWOG1416/Rodler (49) Stage II	Unselected metastatic TNBC. Possibility of having received 1 prior line of systemic treatment in the metastatic phase 335 patients	IV	3 subgroups: A:g BRCAm B: BRCA-like: sBRCAm, HRDscore (Myriad) > 42, BRCA1 promoter hypermethylation, germline mutation in HR genes other than BRCA1/2 C: non-BRCA-like	cisplatin 75 mg/m2/3 weeks (C) vs cisplatin 75 mg/m2/3 weeks + veliparib 300 mgxed/day D1-D14 (VC)	-main: PFS	-PFS A: CV = 6 2 months (95% CI 2 3–9 2) vs C = 6 4 months (HR 0 79 [95% CI 0 38–1 67]; p=0 54) -PFS B: CV = 5.9 months (95% CI 2 3–9 2) vs C = 4.2 months (HR 0 57 [95% CI 0 37–0.88]; p=0 010) -PFS C: CV = 4.0 months (95% CI 2 3–9 2) vs C = 3.0 months (HR 0 89 [95% CI 0 60–1.33]; p=0 57)
Olympia AD/Robson (46) Phase III	HER2- gBRCA1/2m metastatic breast cancers (>49% triple negatives) (No more than 2 metastatic lines previously received) 302 patients	IV		olaparib 300mg x2/day vs investigator’s choice (capecitabine, ribulin, vinorelbine) (CEV)	-main: PFS -secondary: ORR, OS	-PFS olaparib 7.0 months vs CEV 4.2 months (HR 0.58, 95% CI 0.43 - 0.80; P<0.001) -ORR olaparib 59.9% vs CEV 28.8% - OS olaparib 19.3 months vs CEV 17.1 months (HR 0.90, 95% CI 0.66–1.23; *P* = 0.513)
EMBRACA/Litton (47) Phase III	gBRCA1/2m metastatic breast cancer (>40% triple negative) No more than 2 metastatic lines previously received) 431 patients	IV		talazoparib 1 mg/day vs Investigator’s Choice (capecitabine, eribulin, vinorelbine, gemcitabine) (CEVG)	-main: PFS -secondary OS	-PFS talazoparib 8.6 months vs CEVG 5.6 months (HR 0.54; 95% CI, 0.41 to 0.71; P<0.001) - OS talazoparib 19.3 months vs CEVG 19.5 months (HR 0.848; 95% CI, 0.67 to 1.07; P = 0.17)
BROCADE3/Dieras (186) Phase III	HER2- metastatic breast cancers (>47% triple negatives) (No more than 2 metastatic lines previously received) 513 patients	IV		carboplatin (AUC 6) D1 + paclitaxel (80 mg/m² D1 D8 D15/3 weeks) + veliparib (120 mg x2/day D-2 to 5) (CPV) Vs carboplatin (AUC 6) D1 + paclitaxel (80 mg/m² D1 D8 D15/3 weeks) + placebo (CP)	-main: PFS -secondary: OS	-PFS CPV 14.5 months vs CP 12.6 months, HR = 0.71, 95% CI = 0.57 – 0.88, p = 0.0016 -OS CPV 33.5 months vs CP 28.2 months, HR = 0.95, 95% CI = 0.73 – 1.23, p = 0.67
TBCRC009/Isakoff (116) Phase II single arm	Unselected TNBC (1st ^or^ 2^nd^ metastatic line) 43 patients in each subgroup	IV		cisplatin (75 mg/m2) or carboplatin (AUC 6)/3 weeks	-main: ORR	- Overall ORR = 25.6% cisplatin ORR = 32.6% carboplatin ORR = 18.7% -HRD LOH/LST score was higher among BRCA1wt responders than among BRCA1wt non-responders (12.7 vs. 5.1; p = 0.032)
Yardley 2018 (187) Stage II	Unselected TNBC (1st metastatic line) 191 patients	IV		nab-Paclitaxel 125 mg/m2 + carboplatin AUC2 D1 D8/3 weeks (NC) vs nab-Paclitaxel 125 mg/m2 + gemcitabine 1g/m2 D1 D8/3 weeks (NG) vs carboplatin AUC2 D1 D8 + gemcitabine 1g/m2 D1 D8/3 weeks (CG)	-main: PFS -secondary: OS, ORR	-PFS: *NC 8.3 months vs PG 5.5 months, HR 0.59; 95% CI, 0.38–0.92; P = 0.02 *NC 8.3 months vs CG 6.0 months, HR 0.58; 95% CI, 0.37–0.90; P = 0.02 -OS: *NC 16.8 months vs PG12.1 months, HR 0.73; 95% CI, 0.47–1.13; P = 0.16 *NC 16.8 months vs CG 12.6 months; HR 0.80; 95% CI, 0.52–1.22; p = 0.29 -ORR: *NC: 73% *NG: 39% *CG: 44%
O’Shaugnessy (188) Phase III	Unselected metastatic TNBC 519 patients	IV		carboplatin AUC2 + gemcitabine 1g/m2 D1 D8 (GC) vs same treatment + iniparib 5.6 mg/kg D1 D4 D8 D11 (CGI)	-main: PFS and OS	No difference in OS (CG 11.1 vs CGI 11.8 months, HR 0.88; 95% CI, 0.69 to 1.12; P = 0.28) or PFS (CG 4.1 months vs CGI 5.1 months, HR 0.79; 95% CI, 0.65 to 0.98; P=0.27)
TBCSG006/Zhang (50) Phase III	Unselected TNBC (1st metastatic line) 236 patients	IV	A: HRD group = ATM, BARD1, BRCA1, BRCA2, BRIP1, CDH1, CHEK1, CHEK2, FANCA, FANCC, FANCD2, FANCE, FANCF, FANCG, MLH1, MRE11A, MSH2, MSH6, MUTYH, NBN, PALB2, PMS2, PTEN, RAD50, RAD51C, RAD51D, STK11 and TP53 germline mutation B: gBRCA1/2 m group C: HRD group without gBRCA1/2m D: HRP group	cisplatin 75 mg/m² D1 + gemcitabine 1250 mg/m² D1-D8 (CG) vs paclitaxel 175 mg/m² D1 + gemcitabine 1250 mg/m² D-D8 (PG) every 3 weeks for a maximum of 8 cycles	-main: PFS -secondary: OS, ORR	*Overall population: -PFS: CG 7.73 months, 95% CI 6.46–9.00 vs PG 6.07 months, 95% CI 5.32–6.8; P = 0.005. -OS: no significant difference *Analysis in subgroups: -group A: better ORR and PFS in the CG arm than in the PG arm (71.9% vs 38.7%, P = 0.008; 10.37 vs 4.30 months, P = 0.011). -group B: better ORR and PFS in the CG arm than in the PG arm (83.3% vs. 37.5%, P=0.086; 8.90 vs 3.20 months, P = 0.0459). -group C: better ORR and PFS in the CG arm than in the PG arm (70.4% vs. 37.0%, P=0.014; 10.37 vs 3.53 months, P = 0.022). -group D: no difference in ORR and PFS.
TNT/Tutt (48) Phase III	Unselected TNBC (1st metastatic line) 188 patients in each group	IV	A: gBRCAm (n=43) B: HRD Myriad (n=81) C: HRD Myriad or gBRCAm (n = 86)	carboplatin AUC6/3 weeks vs taxotere 100mg/m2/3 weeks 6 rounds	-main: ORR -secondary: PFS and OS	- Overall ORR: carboplatin 31.4% (59/188) vs taxotere 34.0% (64/188); *P* = 0.66 - cohort A ORR: carboplatin 68% vs taxotere 33%; *P* = 0.01 - cohort B ORR: carboplatin 38.2% (13/34) vs taxotere 40.4% (19/47); ns -cohort C ORR: carboplatin 44.7% (17/38) vs taxotere 39.6% (19/48); ns -PFS: carboplatin 3.1 months (95% CI, 2.4–4.2), vs taxotere 4.4 months (95% CI, 4.1–5.1), ns -OS: carboplatin 12.8 months (95% CI, 10.6–15.3) vs taxotere 12.0 months (95% CI, 10.2–13.0); ns

In the neo-adjuvant phase, the addition of Carboplatin to standard chemotherapy based on anthracyclines and taxanes in the treatment of unselected TNBC seems to improve the pathological complete response (pCR) rate with an inconsistent impact in terms of disease-free survival and no significance in terms of overall survival in phase III trials (43, 181, 182). Replacing anthracyclines with a platinum salt also appears to yield comparable pCR rates in this setting (27, 177). Notably, platinum–taxane regimens may even achieve higher pCR rates than anthracycline–taxane combinations, particularly in the BL1 and BL2 subtypes, while similar pCR rates are observed in the M subtype (37–39). However, these comparisons are derived from different studies and should therefore be interpreted with caution. Taken together, these findings nevertheless suggest that TNBC Lehmann molecular subtypes encompassed within the basal-like intrinsic subtype may derive greater benefit from platinum–taxane regimens than from anthracycline–taxane combinations in the localized setting. This may be explained by the high prevalence of HRD in basal-like cancers, as several studies have shown that response to platinum salts in the early setting appears to be associated with HRD status, although this relationship is not consistently observed across studies (40–43). Similarly, the benefit of PARP inhibitors in the early setting is well established in patients with germline BRCA1/2 mutation (173, 174) and is further suggested by phase II studies in other HRD contexts (40, 107). Importantly, the current standard of care in the neoadjuvant setting for stage II or higher TNBC is the addition of pembrolizumab to a chemotherapy backbone combining carboplatin, paclitaxel and anthracyclines, as established in the KEYNOTE-522 trial (189). In this study, the pCR rate reached 64.8%, confirming the high efficacy of this regimen. In this context, the favorable response of tumors presumed to be basal-like (BL1, BL2, and M subtypes) to carboplatin-based regimens may open the door to treatment de-escalation strategies—potentially omitting anthracyclines—in selected TNBC Lehmann subtypes, basal-like, or HRD tumors, with the aim of reducing treatment-related toxicity while maintaining efficacy. However, large-scale prospective studies are required to confirm this hypothesis.

In the metastatic setting, the relationship between HRD and response to platinum salts or PARP inhibitors appears less consistent. A clear predictive value has been established in tumors harboring germline or somatic BRCA1/2 mutations (45–48), as well as in the case of germline PALB2 mutations (45). Beyond these alterations, the clinical relevance of other HRD-related gene mutations remains uncertain, with conflicting data: while some studies (50) suggest improvements in objective response rate and progression-free survival, others (45) do not demonstrate clinically meaningful activity. More broadly, outcomes in HRD-defined but BRCA-wild-type tumors have been disappointing. Thus, carboplatin monotherapy has shown similar response rates to docetaxel in the first-line metastatic setting, with superior efficacy restricted to patients with germline BRCA1/2 mutations, but not in HRD-positive tumors as defined by genomic scar Myriad assay (48). Similarly, the benefit of combining PARP inhibitors with platinum-based chemotherapy remains inconsistent across studies (49, 186, 188), although a signal of activity in “BRCA-like” (sBRCAm, HRDscore (Myriad) > 42, BRCA1 promoter hypermethylation, germline mutation in HR genes other than BRCA1/2) populations cannot be excluded (49). While PARP inhibitors as monotherapy significantly improve progression-free survival in germline BRCA1/2-mutated breast cancers (46, 47), their efficacy in sporadic TNBC remains limited (183).

**Figure 3:**
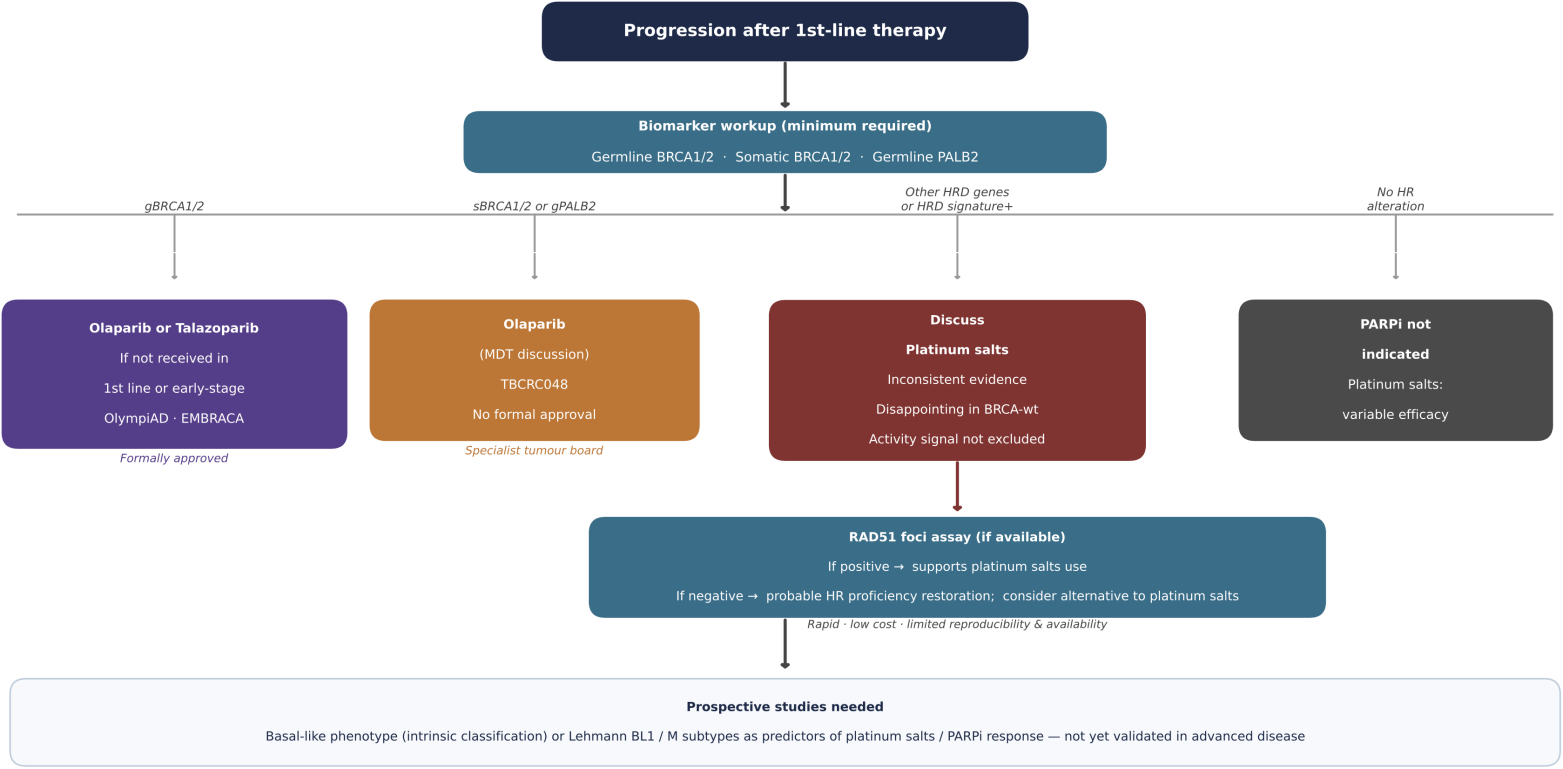
Repositioning algorithm for PARP inhibitors and platinum salts beyond first-line treatment of metastatic TNBC, according to putative or established predictive biomarkers of response.

From a practical standpoint, recent studies have highlighted the growing role of antibody–drug conjugates in the TNBC first-line metastatic setting, including sacituzumab govitecan (as monotherapy or in combination with pembrolizumab in PD-L1 CPS ≥10 tumors) (190, 191) and datopotamab deruxtecan (192). In this evolving landscape, the optimal positioning of carboplatin and PARP inhibitors in subsequent lines remains to be defined (**Figure 3**). Based on the OlympiAD and EMBRACA trials, olaparib and talazoparib can reasonably be considered from the second metastatic line onward in patients with germline BRCA1/2 mutations who have not previously received these agents (46, 47). Their use in tumors harboring somatic BRCA1/2 mutations or germline PALB2 mutations may also be discussed in specialized multidisciplinary tumor boards, based on TBCRC048 (45), although no formal approval exists in these indications. In contrast, for other HRD-related gene alterations or genomic HRD signatures not involving BRCA1/2 or PALB2, current evidence does not support routine use of PARP inhibitors without additional selection criteria. Carboplatin remains an approved option in this setting and may be considered beyond the first line; however, its efficacy appears variable, likely reflecting the emergence of resistance mechanisms, including restoration of homologous recombination proficiency. In this context, functional assessment of homologous recombination—such as RAD51 foci assays—could represent a promising strategy to better select patients, as it provides a real-time evaluation of HR functionality with relatively low cost and rapid turnaround. Nevertheless, its clinical implementation is currently limited by issues of reproducibility and restricted availability. Alternative selection approaches based on basal-like phenotype (by immunohistochemistry or gene expression profiling such as PAM50) or Lehmann molecular subtypes identification could also be explored prospectively, although these methods do not directly assess functional HR status and may face practical limitations related to cost and accessibility.

Furthermore, as a subset of breast cancers outside the TNBC category may exhibit basal-like features and/or HRD, the question of extending such biomarker testing prior to platinum or PARP inhibitor therapy—both in early and metastatic settings—also warrants consideration.

## Discussion

BLBC represent an aggressive subtype of breast cancer, initially defined in the early 2000s based on gene expression profiling within the intrinsic classification (1–3). Although surrogate definitions based on IHC were subsequently developed (62), BLBC accounts for approximately 17–37% of breast cancers using intrinsic classification (3) and around 16% when defined by IHC-based approaches (52, 193). From the outset, the clinical, biological, and molecular similarities between BRCA1-mutated tumors and sporadic BLBC led to the hypothesis that HRD could play a central role in the biology of these tumors, beyond germline BRCA1 alterations.

Accumulating evidence supports this hypothesis, although with important nuances. Despite heterogeneity in definitions and methodologies across studies, a substantial proportion of BLBC display features consistent with HRD. The mechanisms underlying this phenotype are diverse and often mutually exclusive (18). They include germline (approximately 12%) and somatic (approximately 9%) mutations in BRCA1/2 genes, with a predominance of BRCA1 alterations (20), as well as epigenetic mechanisms such as BRCA1 promoter hypermethylation (14–34%) and RAD51C promoter hypermethylation (around 4%) (19, 20, 23). Overexpression of ID4, observed in 37.5–50% of cases, has also been implicated in the repression of BRCA1 expression (21). Beyond these alterations, additional mechanisms—including rare inactivating alterations in other homologous recombination genes or transcriptional and post-transcriptional repression—could further contribute to HRD, suggesting the possible existence of non-mutational origins of this phenotype in a substantial subset of BLBC. The causes of these non-mutational alterations remain incompletely understood. Emerging data suggest that tumor microenvironment–derived factors may play a role. For instance, cytokines such as TGFβ have been shown *in vitro* to induce transcriptional repression of several homologous recombination genes, notably through the involvement of the miR-181 family (25). Such mechanisms may promote the emergence of a “BRCAness” phenotype characterized by genomic instability and increased sensitivity to DNA-damaging agents. These observations raise the possibility that, in basal-like carcinogenesis, microenvironmental stresses may either substitute for or cooperate with canonical genetic alterations to induce HRD. A better understanding of these processes could open new avenues for therapeutic intervention. Despite this strong biological rationale and theranostic interest, several important limitations must be acknowledged. A significant proportion of available data is derived from triple-negative breast cancer (TNBC) cohorts, in which approximately 80% of tumors are basal-like (4, 5, 115), while the remaining ~20% correspond to non–basal-like subtypes such as LAR and MSL, which are less strongly associated with HRD (28, 177). Conversely, a subset of non–triple-negative tumors may transcriptionally correspond to basal-like and HRD phenotypes. These observations suggest that intrinsic molecular classification may provide a more relevant framework than TNBC status alone for studying HRD biology and therapeutic sensitivity. In addition, functional assays assessing homologous recombination activity, such as RAD51 nuclear foci formation, consistently demonstrate that only a subset of basal-like tumors exhibit true HRD (24, 29). This has been observed both in basal-like cell line models (24) and in patient-derived xenografts (29), where approximately half of basal-like models were functionally HR-deficient despite higher rates of genomic HRD. This discordance reflects the fact that genomic scar–based approaches capture historical HRD and may persist even after restoration of homologous recombination function, for example through reversion mutations in HR genes or loss of factors such as 53BP1 (194). Furthermore, intratumoral heterogeneity and clonal evolution under treatment pressure may lead to the coexistence of HR-deficient and HR-proficient cell populations. Additional resistance mechanisms, including increased drug efflux and enrichment in cancer stem cell populations, further complicate the relationship between HRD and therapeutic response. From a clinical perspective, therapeutic targeting of HRD in breast cancer has primarily relied on platinum-based chemotherapy and PARP inhibitors. However, unlike in ovarian cancer (34–36), HRD has not yet been prospectively validated as a predictive biomarker in this setting. While TNBCs may show meaningful responses to these agents in selected contexts—particularly in early-stage disease—outcomes in the metastatic setting remain more heterogeneous and often limited (**Table 5**), likely reflecting the emergence of resistance mechanisms and restoration of homologous recombination proficiency. In this context, several complementary strategies could be explored to refine patient selection, including intrinsic subtype classification, molecular subtyping according to Lehmann, associated with genomic or functional assessment of HRD. Among these, real-time functional assays such as RAD51-based approaches are particularly attractive from a biological standpoint, but their clinical implementation raises important challenges in terms of standardization, and reproducibility. At present, these approaches should therefore be considered as hypotheses to be tested prospectively rather than tools ready for routine clinical decision-making. Despite these limitations, focusing on BLBC rather than TNBC offers several conceptual and practical advantages. Intrinsic molecular classification allows the identification of a biologically more homogeneous disease entity and provides a more integrated understanding of tumor biology, disease behavior, and outcome patterns. It may also enhance prognostic stratification by reducing the confounding effect of non–basal-like TNBC subtypes. From a translational perspective, BLBC constitutes a more coherent framework for identifying therapeutic vulnerabilities and developing targeted strategies, not only in the context of HRD but also beyond. In addition, it enables the definition of more homogeneous patient populations for future clinical trials, thereby increasing the likelihood of detecting clinically meaningful treatment effects. Distinguishing BLBC from TNBC, refining TNBC classification using Lehmann subtypes, and integrating HRD assessment may therefore all carry potential clinical relevance. In early-stage disease, improved identification of basal-like tumors and their HRD status could contribute to treatment de-escalation strategies in selected patients. In the metastatic setting, intrinsic subtype classification, Lehmann subtyping, and HRD assessment—particularly when integrating functional readouts—may represent complementary approaches to better understand therapeutic sensitivity to DNA-damaging agents, including platinum salts. However, their respective predictive value remains to be established in prospective studies, and their integration into clinical practice will depend on demonstrating added value over existing clinico-pathological criteria. To clarify these distinctions, the key differences between TNBC and BLBC, as discussed throughout this manuscript, are summarized in **Table 6**.

**Table 6:** Comparison between triple-negative breast cancer (TNBC) and basal-like breast cancer (BLBC).

Feature	TNBC	BLBC
Definition	Immunohistochemical (ER-, PR-, HER2-)	Intrinsic molecular subtype (gene expression profiling, PAM50)
Prevalence	~15–20% of breast cancers	~17–37% (intrinsic), ~16% (IHC surrogate)
Overlap	~70–80% are basal-like	Majority are TNBC, but some are non-TNBC
Molecular heterogeneity	High (includes BL1, BL2, LAR, M, MSL)	More homogeneous but still heterogeneous
HRD prevalence (genomic)	~41,5–76,9% overall	Enriched (70-80%), particularly in BL1 and M subtypes (>75%))
HRD prevalence (functional, RAD51)	Variable, not systematically assessed	~46–50% in basal-like models
BRCA1 alterations	Present in subset	Enriched
Epigenetic HRD mechanisms	Present but variable	BRCA1 (14–34%), RAD51C (~4%) hypermethylation, ID4 overexpression (37.5–50%)
Non-mutational HRD	Less well characterized	Possible (transcriptional repression, microenvironmental effects)
Clinical relevance of HRD	Used as surrogate in trials	Likely more biologically relevant framework
Therapeutic implications	Platinum, PARP inhibitors (heterogeneous response)	Potentially improved stratification using subtyping + HRDassesment
Limitations	Includes non–HRD-enriched subtypes (LAR, MSL)	Requires transcriptomic classification, limited routine use

## Conclusion

BLBC represent a biologically coherent and HRD-enriched subtype of breast cancer, characterized by a complex interplay of genetic, epigenetic, and microenvironmental mechanisms. While HRD constitutes a key vulnerability in this context, it is not a universal feature, and its clinical exploitation remains limited by biological heterogeneity and the lack of robust, prospectively validated predictive biomarkers. Integrating intrinsic subtype classification with both genomic and functional assessment of homologous recombination offers a promising framework to better capture clinically relevant HRD. However, translating these insights into therapeutic decision-making will require carefully designed prospective studies addressing feasibility, reproducibility, and clinical utility, in order to balance biological relevance with real-world applicability in basal-like breast cancer.
